# Expanding budget space to improve health outcomes in low- and middle-income countries: what role for tax expenditures?

**DOI:** 10.1093/heapol/czaf079

**Published:** 2025-10-22

**Authors:** Abrams M E Tagem, Yann Tapsoba, Hélène Barroy

**Affiliations:** African Tax Administration Forum (ATAF), Pretoria, 4 Daventry Street, Lynnwood Manor, Pretoria 0181, South Africa; Financing Alliance for Health, Nairobi 1185 Mzima Springs Lane, Off Riverside Drive, Nairobi, Kenya; Department for Performance, Financing and Delivery, World Health Organization (WHO), Avenue Appia 20, Geneva 1211, Switzerland

**Keywords:** budget space for health, health outcomes, public financial management, tax expenditures

## Abstract

Recent evidence indicates that budget space for health can be improved through increasing government revenues, expanding the budget’s health share, and improving expenditure efficiency through enhancing public financial management (PFM), with government revenue mobilization being the most substantial. Government revenue mobilization can be achieved by broadening the tax base, a key component of which is the rationalization of tax expenditures. Tax expenditures are preferential tax treatments, relative to a baseline tax regime, intended to achieve specific objectives by providing financial support to specific beneficiaries. They may, however, result in huge revenue losses, which could be otherwise invested in priority sectors, including health. In addition, tax expenditures ultimately exacerbate inequality, while also creating complexities that foster tax avoidance and evasion, all of which contribute to deteriorating health outcomes. In the context of scarce public finances in low- and middle-income countries, rationalizing tax expenditures can create the necessary fiscal space for development. This paper provides a first comprehensive analysis of the ‘health costs’ of tax expenditures by analysing the relationship between tax expenditures and health outcomes, with a focus on under-five and maternal mortality. Using data from 55 developing countries from 2000 to 2022, we find that an increase in tax expenditures leads to higher under-five and maternal mortality, especially in low-income countries. The results are robust to several instrumental variable strategies, alternative measures of tax expenditures, and alternative methods. We also find that PFM, through the quality of public administration, transparency in the public sector, and the efficiency of revenue mobilization, mitigates the corrosive effects of tax expenditures. A key implication of our findings is that understanding the ‘health costs’ of tax expenditures is a necessary precursor to eliminating wasteful tax expenditures, the benefits of which can contribute to expanding the budget space for health and improving health outcomes.

Key messages

- Tax expenditures are deviations from a benchmark tax regime that grant financial support to targeted beneficiaries, which directly reduce government revenues (which could have been invested in the health sector).
- Revenue foregone from tax expenditures are associated with worse child and maternal health outcomes, with the effects stronger on maternal health outcomes.
- Higher public financial management quality, especially the quality of public administration, budget transparency, and efficiency of revenue mobilization, mitigates the negative effects of tax expenditures on health outcomes.
- Tax expenditure reform is a relatively low-hanging fruit for improved domestic revenue mobilization. However, rationalization of tax expenditures requires understanding their costs, and this paper is the first cross-country econometric study to estimate those costs and losses.

## Introduction

### Background

Countries are committed to achieving the Sustainable Development Goals (SDGs) by 2030, including on health (SDG target 3), which consists of improving specifically maternal and under-five mortality. While developing countries have made progress on both mortality indicators, reducing maternal and under-five mortality by 37.4% and 49.6% over 2000–2020, respectively, progress is still needed to meet the targets (World Bank 2024). Funding needs to meet the health-related SDG targets, including universal health coverage (UHC), are significant. Stenberg et al. (2017) reported that additional investments needed to achieve the targets under an ambitious scenario will amount to USD 371 billion by 2030 (USD 58 per capita), while under a less ambitious, ‘progress’ scenario, investments will amount to USD 274 billion by 2030 (USD 41 per capita). These investments will be needed to broadly strengthen health systems, such as by improving health systems infrastructure, expanding human resources for health, and increasing access to essential medicines and vaccines.

United Nations (UN) Financing for Sustainable Development Reports emphasize the role of increased domestic revenue mobilization (DRM) to finance development (and consequently health) outcomes, in recognition of dwindling foreign assistance and the fact that most domestic health spending is financed from domestic revenue sources (Asante et al. 2020). Domestic public funding is essential to finance the investments and interventions needed to increase access to better child and reproductive health services, its salience now being emphasized in the literature (Atim et al. 2020).

Recent evidence shows that governments have several options to expand budget space for health—‘the potential resources budgeted and used for health through the PFM system’: government revenue mobilization, health sector budget prioritization, improving expenditure efficiency, and public financial management (PFM) (Heller 2006, Barroy and Gupta 2021). Empirical evidence shows that government revenue mobilization is the most important pillar, accounting for 70% of the change in government expenditures on health (Barroy et al. 2018). Government revenue is financed mainly through taxation, both direct taxation (i.e. taxes collected directly from individuals and businesses, e.g. personal and corporate income taxes) and indirect taxation (i.e. taxes collected indirectly through consumption, e.g. excise and value-added taxes). In addition, taxes are considered the most progressive means of healthcare financing, i.e. tax-funded healthcare represents a higher share of the income of the rich (Loewenson and Mukumba 2023). The potential for increasing tax revenues in low-income countries (LICs) is significant, with average tax/GDP ratios low and stagnant. Increased taxes can be achieved through ambitious tax reforms, for which an expansion of tax bases is necessary. A core component of tax base expansion is the rationalization of tax expenditures, which has enormous long-run revenue potential of up to 2% of GDP (IMF 2024).

### What are tax expenditures?

Tax expenditures can be defined as deviations from a benchmark tax regime intended to provide financial support to targeted beneficiaries (e.g. individuals, companies, economic, and/or geographical sectors) by ultimately lowering the tax liability of the beneficiaries (IMF 2022). These deviations, which are viewed as an alternative to direct government spending, can be granted through complete exemptions, preferential rates (usually reduced rates), deductions, and credits (Von Haldenwang et al. 2023). Tax expenditures are used to develop specific sectors, increase access to specific goods and services, and attract private investment (ECA 2024), and are mostly split between tax expenditures on income and those on goods and services. They can be embedded in statutory law (as is common in developed countries) or included in investment codes, individual contracts, or even granted by government decree (the latter is common in developing countries). Over recent decades, tax expenditures have proliferated across countries and regions, with the global average of revenue foregone resulting from tax expenditures amounting to 3.8% of GDP and 23% of tax revenue (Von Haldenwang et al. 2023).

### Why analyse the relationship between tax expenditure and health outcomes?

The literature postulates various factors as determinants of health outcomes in developing countries, with economic, social, institutional, and increasingly domestic financing variables particularly salient (see Junior et al. 2023, Tadadjeu et al. 2023, for a review). However, there is no empirical evidence on the effect of tax expenditures on health outcomes in developing countries, although they could be strong drivers or hinderers of maternal and child health outcomes. Tax expenditures result in revenue losses, and their rationalization could serve as a lever to increase resource mobilization for health, potentially contributing to the improvement of health outcomes in developing countries.

In addition, recent research emphasizes the role of PFM, especially in health, in improving health outcomes. For a sample of 42 SSA countries over the period 2005–5018, Tapsoba et al. (2024) showed that a high level of PFM—specifically, strong and predictable budget allocation—is associated with better health outcomes, especially child and maternal health outcomes. Strong PFM quality, especially transparency, is also necessary for the management of tax expenditures. Given that tax expenditures can be granted by various authorities within a country, their proliferation is inevitably prone to abuse through rent-seeking and a general lack of transparency and oversight (Von Haldenwang et al. 2024).

Against this backdrop, this paper provides the first econometric analysis linking tax expenditures to health outcomes in developing countries. The paper seeks to establish a relationship between tax expenditures and the under-five mortality rate and maternal mortality ratio in low- and middle-income countries. In addition, the paper seeks to uncover the importance of PFM in mediating the relationship between tax expenditures and health outcomes. Furthermore, the paper explores heterogeneity in the relationship based on countries’ level of economic development. This paper thereby contributes to the literature on the role of taxes in financing health systems and improving health outcomes, the discussion on budget space for health, and the burgeoning literature on factors eroding DRM.

## Methods

### Conceptual framework

Tax expenditures are provided to meet specific policy objectives, including attracting investment, increasing access to specific goods and services, as well as boosting specific sectors (ECLAC 2022, ECA 2024). The health sector may also benefit from tax expenditures in a bid to prioritize increased access to healthcare (Table 1 provides a summary of various health-related tax expenditures).

**Table 1.** Examples of health-related tax expenditures.

Tax type	Economic function	Policy objective	Beneficiaries	Examples
PIT	Exemptions^a^	Incentivise employment in specific areas; promote savings for future medical expenses	Healthcare providers; healthcare consumers	Exemptions for health workers in rural or underserved areas
	Deductions^b^	Reduce the cost of OOP expenses; encourage employer-provided health insurance	Healthcare consumers (e.g. individuals with high medical expenses, employees)	Expenses for dependent’s healthcare
	Credits^c^	Increase the affordability of health insurance and/or services	Individuals or households	Credits for low-income individuals for health-related expenses or insurance premiums
	Preferential rates^d^	Incentivise employment; promote savings for future medical expenses	Healthcare providers; healthcare consumers	Reduced rates for health specialists (e.g. cardiologists)
CIT	Exemptions	Support NGOs and non-profit hospitals importing medical necessities; domestic pharmaceutical companies	Healthcare providers (both public and non-profit)	Tax holidays for new clinics or diagnostic centres, or profits from sales of essential medicines
	Deductions	Support health investment in underserved and/or rural areas	Healthcare providers (both public and non-profit)	Deductions for infrastructure investments in underserved areas
	Credits	Support research and development (R&D) in healthcare	Pharmaceutical producers; healthcare providers	Credits for R&D in medicine, vaccines, or medical devices
	Deferrals^e^	Boost domestic production of key medical equipment and products	Pharmaceutical producers; healthcare providers (e.g. hospitals)	Accelerated depreciation on capital investments by pharmaceuticals
VAT	Exemptions	Reduce the costs of healthcare services and medical products	Healthcare consumers; healthcare providers (non-profit and public)	Exemptions on essential medicines, medical equipment, health-related consumables, and public healthcare services
	Reduced rates	Increase the affordability of health products	Healthcare consumers, pharmaceutical consumers	Reduced rates on prescription drugs, sanitary products, and children’s vaccines
Excise taxes	Exemptions	Reduce the costs of specific health-related goods	Manufacturers	Waivers on inputs for producing vaccines or life-saving drugs
Customs duties	Exemptions	Lower costs of imported medical equipment and medicines	Manufacturers (importers), healthcare providers	Duty-free imports of essential medical equipment
	Reduced rates	Facilitate the availability of critical health-related imports	Hospitals, patients	Raw materials for pharmaceutical production

The table provides examples of common health-related tax expenditures broken down by tax type, economic function, policy objective, and beneficiaries.

PIT, personal income tax; CIT, corporate income tax; VAT, value-added tax.

^a^Exclusions from the tax base.

^b^Amounts deducted from the tax base before applying the statutory tax rate.

^c^Amounts deducted from tax liability.

^d^Different (typically reduced) tax rates.

^e^Delays in paying the tax liability.

Health-related tax expenditures can include tax holidays that provide complete exemption from corporate income tax (CIT) for domestic companies producing essential drugs and hospital equipment, value-added tax (VAT) exemptions on supplies of basic necessities, VAT exemptions and reduced rates on medical products, customs duty exemptions on imported medical goods and inputs required to produce drugs (especially those used by domestic pharmaceutical companies), and deductions or credits to reduce the cost of private out-of-pocket (OOP) health expenditures (OECD 2020a, 2020b). These tax expenditures may contribute to reduced costs of healthcare, reduced inequality in health service provision, and improved health outcomes (OECD 2020a).

Impact evaluation of tax expenditures would determine their ultimate impact on stated objectives, since some tax expenditures may present advantages over direct spending outlays (especially in countries with limited revenues to fund redistribution through social assistance programmes). Properly designed health-related tax expenditures that meet their social and economic objectives ease compliance and can contribute to improved population health outcomes. However, in developing countries, tax expenditures are typically overused to correct for other deficiencies in the tax expenditure system, making them inadequate in achieving their stated objectives. The most common deficiencies result from low tax ratios (hence, less fiscal space for general and/or social sector spending), huge dependence on regressive indirect taxes that constrain the redistributive capacity of governments, and a lack of targeting in direct transfers and other welfare benefit systems (World Bank 2022). Thus, tax expenditures are more likely to adversely impact health outcomes—i.e. higher revenue foregone worsening health outcomes, and the effects operate in three ways.

First, the primary mechanism through which tax expenditures influence health outcomes is a direct fiscal cost (or loss), i.e. a direct reduction of government revenue. Tax expenditures narrow the tax base and result in considerable revenue losses (Junior et al. 2023, Masiya et al. 2024), especially in developing countries with large informal and agricultural sectors (World Bank 2022). Once the tax expenditure is provided (an implicit subsidy), the government has less money, which could be invested in health and other sectors. A core aspect of the fiscal system that reinforces the direct fiscal cost of tax expenditures is the political vulnerability to keep granting exemptions and/or reduced rates: an exemption for one sector or taxpayer creates pressure to grant exemptions to similar taxpayers in other sectors (i.e. ‘exemption creep’) and increased pressure for other goods and services to also benefit from lower rates (i.e. ‘reduced rate creep’) (Cnossen 2015). The only way to secure government revenue under such narrow tax bases is to increase marginal tax rates on non-beneficiary taxpayers than would otherwise be done in the absence of tax expenditures, with potentially distortive economic effects.

Second, tax expenditures create complexities in administering different rates and exemptions, fostering tax avoidance and evasion (as well as other illicit financial flows), exacerbating their corrosive effects on public finances (Von Haldenwang et al. 2024). This results in significant revenue leakage (especially for CIT and VAT, two key taxes in developing countries), which has negative effects on social outcomes. Ortega et al. (2018, 2020) showed that a higher ratio of illicit financial flows to total trade (demonstrating the dominance of the former) reduces government revenues and ultimately has a negative effect on maternal health services and immunization rates in developing countries. Etter-Phoya et al. (2023) showed that the larger fiscal space from curtailing tax avoidance is associated with improved access to water and sanitation for children in Malawi.

Third, tax expenditures jeopardize the progressivity and fairness of tax systems and create economic distortions, resulting in ultimately regressive tax policies that exacerbate inequality. Two features of tax expenditure design compromise their effectiveness: tax incidence and targeting of beneficiaries. Regarding incidence, reduced rates and exemptions benefit consumers only through reduced consumer prices, but the reduction in tax rates can also be captured as higher profit margins for intermediaries and retailers, rather than through lower prices for consumers. Reduced rates can also lead to substitution effects (i.e. consumers switching from the product altogether), ultimately bungling the rate differentiation policy. Regarding beneficiaries, while tax expenditures on basic necessities reduce the effective tax rate on consumption for low-income individuals, they unintendedly confer large subsidies to higher-income individuals since the latter spend more in absolute terms, exacerbating income and consumption inequality (Cnossen 2015). This increased inequality contributes to the worsening of health outcomes (Acheampong and Opoku 2024). In addition, since higher-income individuals are likely to have formal employment and/or resources to invest in private healthcare, they are more likely to benefit from such PIT-related tax expenditures.

**Figure 1.**
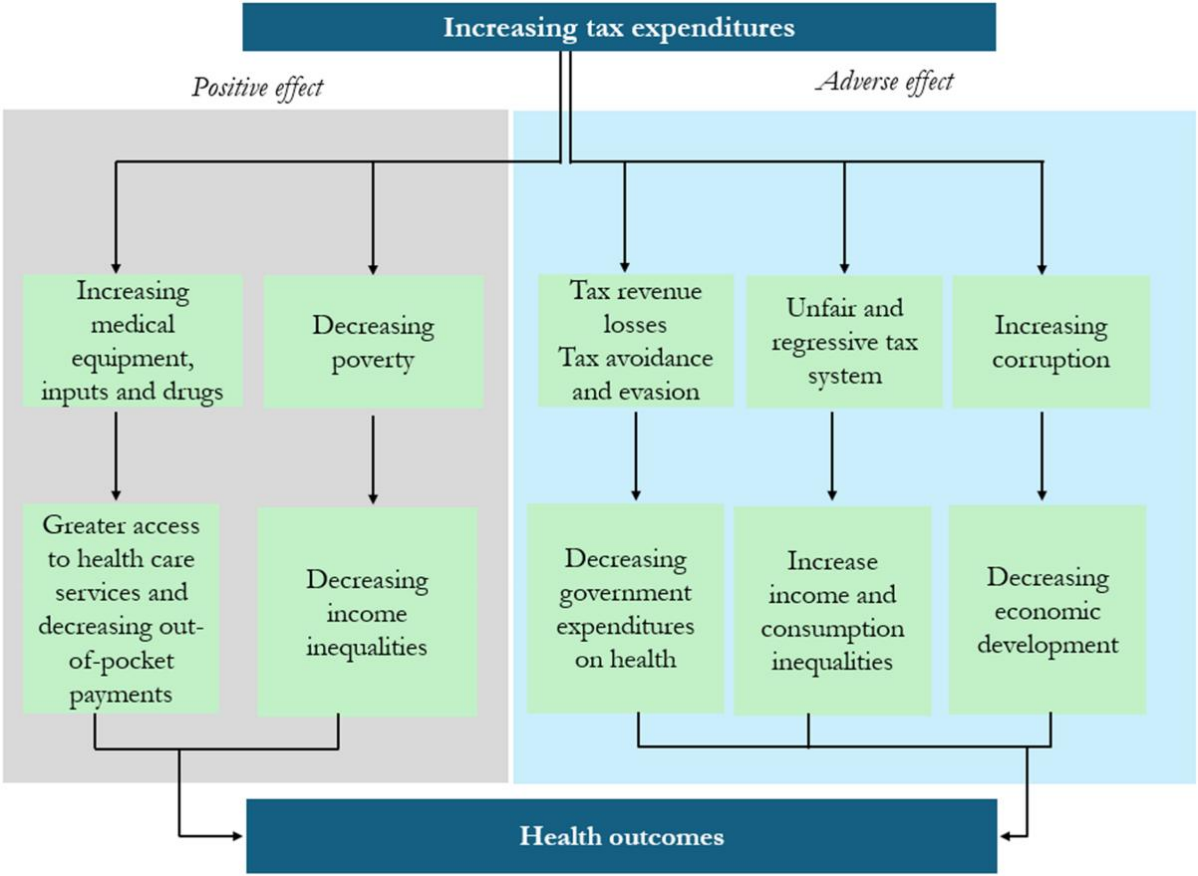
Connecting tax expenditures to health outcomes.

The transmission channels discussed above may appear long and complex, especially with the use of mortality indicators, which are standard in the cross-country health economics literature. However, the channels are very plausible in a developing country context, and Fig. 1 provides a conceptual illustration that summarizes these key channels through which tax expenditures influence health outcomes.

### Data

The sample is an unbalanced panel of 55 developing countries over 2000–2022. The list of countries and summary statistics are reported in the Supplementary Materials (SM) (Supplementary Appendix A and Table A1, respectively), Supplementary Table A2 provides a summary of general tax expenditures, and Supplementary Table A3 provides a correlation matrix for included variables. The focus on low- and middle-income countries is for two key reasons. First, these countries simultaneously have low average tax ratios (which inevitably limit government spending and redistribution) and high levels of tax expenditure. These twin issues, in addition to the standard constraints they face in raising revenues, make rationalizing tax expenditures a key strategy for generating revenue to finance population health. Second, these countries use tax expenditures mainly to redistribute income, incentivize businesses, and attract investment (while high-income countries mostly use them to boost social welfare), resulting in higher shares of VAT and CIT-related tax expenditures compared with PIT-related ones (Von Haldenwang et al. 2023, ECA 2024). Since these countries also depend more on VAT and CIT revenue relative to PIT (the latter, in part, due to large informal sectors), huge tax expenditures (especially tax exemptions and holidays) on the most important taxes are counterproductive.

The dependent variables included are the under-five mortality rate and the maternal mortality ratio. The under-five mortality rate measures the probability that, per 1000 births, a baby will die before reaching 5 years old. It is a measure of the strength of a country’s health sector, as well as its sensitivity to changes in socio-economic development and basic living conditions (Bishai et al. 2016). The maternal mortality ratio measures the number of women who die from pregnancy-related complications during pregnancy or 42 days of pregnancy termination per 100 000 live births. Data on both variables are obtained from the World Development Indicators (WDI) database.

The independent variable of primary interest is revenue foregone, which is an *ex-post* estimate of the direct fiscal cost of the specific tax expenditure provision under consideration, relative to the benchmark tax regime, which does not have such provisions (IMF 2019). The measure of revenue foregone comprises revenue foregone from all major taxes and includes all possible tax expenditure provisions. We obtain revenue foregone in local currency units (LCU) from the Global Tax Expenditures Database (GTED 2024). We convert the revenue foregone (LCU) variable to per capita terms (to ascertain the health impact of revenue losses on an individual) and purchasing power parity (PPP) to incorporate cost-of-living differences, allow for cross-country comparability, and better capture resource availability (i.e. what the country can actually afford and implement with its resources). While all included countries estimate the cost of tax expenditures using the revenue foregone method (thus ensuring relative consistency), the heterogeneity across countries in defining the benchmark tax system suggests that our results should be interpreted as comparative tendencies in effects across countries.

Control variables are chosen following the literature (Tadadjeu et al. 2023, Acheampong and Tetteh 2024). The baseline model includes GDP per capita (constant PPP), domestic health expenditure per capita (constant PPP)—which does not include on budget development assistance for health (DAH), foreign direct investment outflows per capita (USD), household final consumption per capita (USD), out-of-pocket payments (percentage of current health expenditure), and external health expenditures on health per capita (constant PPP), which includes on-and-off-budget DAH. The variables are obtained from the WDI and Global Health Expenditure Databases.

GDP per capita is included as a proxy for the overall wealth of a country. As countries expand economically, mortality reduces through an increase in government revenue, government spending, and the provision of health services, as well as through increased household income (Hall et al. 2021). Government health expenditure is a key determinant of population health, with higher values implying a larger budget allocated to the health sector and possibly a strong political commitment towards improving health outcomes. Nevertheless, in the context of high inefficiency in spending and inefficient health systems, higher health spending may not be associated with improved health outcomes (Kabongo and Mbonigaba 2024). The impact of FDI per capita is theoretically ambiguous. On the one hand, FDI is associated with productivity increases, economic growth, and an expansion of key tax bases, all of which generate improvements in health through technological progress, learning-by-doing, and investments. On the other hand, multinational corporations are the primary beneficiaries of tax incentives and exemptions, creating revenue losses perpetuated by tax avoidance, adversely affecting population health outcomes (Etter-Phoya et al. 2023).

Higher household consumption per capita is meant to improve health outcomes, particularly through the effects of higher disposable income. Higher-income households can afford to expand consumption on non-health items without necessarily compromising their ability to spend on their health needs. They also tend to be able to afford better healthcare services, which increases their chances of survival, hence have lower child and maternal mortality ratios. Out-of-pocket payments (% current health expenditure) are official payments made directly to the healthcare providers and institutions (usually charges for services intended to be free), as well as indirect costs linked to transportation, housing, and feeding. An increase in the share of such expenditures, beyond a certain threshold of disposable income (typically greater than 40%), leads to financial hardship and under-utilization of healthcare (Khan et al. 2017). External health expenditure (DAH) is incorporated into the model to control for the health sector dependency on external funding, especially in LICs, where it contributes to health service delivery and health system strengthening, leading to health improvement (Bendavid and Bhattacharya 2014).

Institutions are included only to gauge their mediating impact on tax expenditures. Specifically, we focus on measures of PFM quality since it has been shown that improvements in PFM are crucial to expanding budget space for health (Barroy and Gupta 2021). We use three Country Policy and Institutional Assessments (CPIA) indices as proxies for PFM quality, which we posit to have a more direct link with tax expenditures, obtained from the World Bank CPIA database. The variables, which range from zero (low) to six (high) respectively, approximate: (i) the quality of public administration; (ii) transparency, accountability, and corruption control in the public sector; and (iii) the efficiency of revenue mobilization. Data cover the period 2005–2022 for only 26 of the 55 countries chosen, reducing the sample.

### Baseline regression analysis

To estimate the impact of tax expenditures on health outcomes, we specify the following linear model:


(1) $$Y_{it}=\alpha_{i}+\lambda_{t}+\beta \cdot RF_{it}+\sum_{k=1}^{n}\delta_{k}\cdot X_{itk}+\varepsilon_{it}$$

where *i* = 1, …, *N* (55 countries), *t* = 1, …, *T* (23 years), and all the variables are in natural logarithms (permitting estimation of elasticities). $Y_{it}$ is the specific measure of population health outcomes in country *i* at time *t*, $RF_{it}$ represents revenue foregone per capita PPP, and $X_{it}$ represents the set of controls described above. All models include a full set of fixed effects, $\alpha_{i}$, to incorporate country-specific time-invariant characteristics (e.g. natural resources and geography) and year fixed effects, $\lambda_{t}$, to account for global developments which affect countries similarly. $\varepsilon_{i,t}$ is the idiosyncratic error term.

### Instrumental variable approach

Contemporaneous reverse causality and simultaneity may bias the baseline analysis. Since we use an aggregate measure of revenue foregone that includes special provisions with various objectives, the variable does include revenue foregone from health-related tax expenditures, which are meant to directly influence health outcomes (see the Methods section). In such cases, the current level of health outcomes may influence spending through health-related tax expenditures. Additionally, poor health outcomes may result in an adjustment of tax expenditures to counter them. Furthermore, an independent variable (such as corruption and/or PFM quality) may determine both population health outcomes and the level of tax expenditures. For example, corruption can reduce health outcomes directly by reducing access to healthcare and eroding service quality, while it can also weaken PFM, with negative consequences on health outcomes (Bukari et al. 2024). In addition, low budget transparency can result in the most inefficient tax expenditures being awarded (e.g. poorly targeted discretionary tax expenditures), exacerbating their negative effects.

Instrumental variable (IV) techniques can circumvent the endogeneity problem by using only the part of the variation in the tax expenditure variable that is uncorrelated with the error term: i.e. finding an external instrument that predicts tax expenditures but is unrelated to health outcomes. The novelty of the research question means that there are no known external instruments for tax expenditures, so we use the lags of revenue forgeone per capita (two lags, used together) as external instruments for revenue foregone (The use of lags as external instruments for endogenous variables is not uncommon in the health literature. For example, Acheampong and Tetteh (2024) and Banerjee et al. (2023) use lags of financial inclusion to assess its impact on population health, Gopalan and Rajan (2016) use lags of sector aid to assess its impact on access to water supply and sanitation.). Conceptually, lags of revenue foregone could be adequate external instruments since the estimation of revenue foregone depends crucially on the definition of a benchmark tax regime, which has implications for the definition, classification, and costing of tax expenditures (Aliu and Redonda 2024). The core features of a benchmark tax regime, i.e. the income and consumption tax bases, as well as the broad rate structures, do not change significantly year-on-year. This makes lagged tax expenditures a strong predictor of current tax expenditures. We use two lags to preserve degrees of freedom, and diagnostic tests, specifically relevance and exogeneity, confirm the validity of the instruments. We complement the above by using internally generated instruments based on heteroskedasticity in the error terms (Lewbel 2012), where identification is achieved by using regressors that are uncorrelated with the product of heteroskedastic errors.

### Interaction regression analysis

Using the same econometric approach described in equation (1), the following interaction term model has been specified:


(2) $$\begin{array}{rl} Y_{it}\;= & \alpha_{i}+\lambda_{t}+\beta \cdot RF_{it}+\mu \cdot \mathrm{PF}M_{it}+\gamma \cdot RF_{it}\cdot \mathrm{PF}M_{it}\\ & +\sum_{k=1}^{n}\delta_{k}\cdot X_{itk}+\varepsilon_{it} \end{array}$$

where $\mathrm{PF}M_{it}$ have been proxied through a set of key tax-related CPIA indexes as described above. The quality of public administration assesses the extent to which civilian central government staff is structured to design and implement government policy and deliver services effectively. Transparency, accountability, and corruption control in the public sector assesses the extent to which the executive can be held accountable for its use of funds and for the results of its actions by the electorate and by the legislature and judiciary, as well as the extent to which public employees within the executive are required to account for administrative decisions, use of resources and results obtained. The efficiency of revenue mobilization assesses the overall pattern of revenue mobilization, not only the *de facto* tax structure, but also revenue from all sources as actually collected.

### Robustness analysis

We performed five sensitivity analyses to ascertain the robustness of the primary findings. First, we use two alternative measures of revenue foregone, as reported by the GTED: revenue foregone (% total tax revenue) and revenue foregone (% GDP). Second, we split the sample based on level of development, distinguishing between upper middle-income countries (UMICs) and a combination of low-income and lower middle-income countries (LLMICs), the former comprising 21 countries and the latter 34. Third, we add control variables typically included in cross-country regressions that posit the determinants of health to include proximate primary healthcare factors like healthcare, sanitation, access to clean water, and education (Bishai et al. 2016). Data availability (i.e. coverage across countries and over time) restricts the choice of and motivation for including some variables, acknowledging that some of these variables are weak proxies. Nevertheless, all the selected variables have been used in the literature, and all of them are strongly correlated with other proxies meant to capture the same concept. The variables include: hospital bed supply, share of people using at least basic sanitation services, the 1-year lagged gross secondary enrolment rate (to account for the time inertia in its effect on health outcomes), governance index (The governance index was computed through principal components analysis (PCA) using the first principal and including the following variables: government effectiveness, voice and accountability, political stability, regulatory quality, rule of law, control of corruption, and the absence of violence or terrorism.), and population density. To account for the level of health prioritization in government budgets, we replace domestic health expenditure per capita with government health expenditure (% domestic government expenditure). For the under-five mortality rate only, additional variables also include prevalence of stunting in children under five and the child immunization index (The immunization index was also computed through PCA and including the following variables: immunization coverage against BCG, DPT, Hepatitis B, Measles, Poliomyelitis, and Haemophilus influenzae type B.). All these variables were obtained from the WDI and World Governance Indicators. Fourth, we test the robustness of our primary findings to three alternative methods: random effects (a direct alternative to fixed effects), feasible generalized least squares (to account for potential heteroskedasticity and serial correlation in the baseline model), and the Driscoll and Kraay (1998) estimator (to incorporate cross-section dependence in both tax and health data). Finally, we introduced threshold (broadly non-linear) effects of revenue foregone on mortality indicators, using two approaches: a quadratic form of revenue foregone and a sample split of the revenue foregone variable based on its median.

## Results

### Baseline findings

Table 2 reports baseline findings. Columns [1]–[3] are respectively for revenue foregone per capita PPP (baseline estimates), revenue foregone (% GDP), and revenue foregone (% tax revenue), and the under-five mortality rate as the dependent variable, columns [4]–[6] reported analogously with the maternal mortality ratio as the dependent variable. The baseline results show that an increase in tax expenditures is associated with increased under-five and maternal mortality. A one percentage increase in revenue foregone is associated with a 0.025% increase in the under-five mortality rate and a 0.051% increase in the maternal mortality ratio. Using the estimated coefficients from Table 2 and the descriptive statistics in Supplementary Table A1, the results show that the effect of tax expenditures on maternal mortality is larger than that on the under-five mortality rate (We obtain the size of the estimated coefficients by dividing the coefficient on the under-five mortality rate by the mean of under-five mortality in the sample, do it analogously for the maternal mortality ratio, and compare both.). In summary, the evidence shows that increasing tax expenditures jeopardizes population health improvement by increasing infant and maternal mortality.

**Table 2.** Tax expenditures and health outcomes, global sample.

	U5MR			MMR		
	[1]	[2]	[3]	[4]	[5]	[6]
RF per capita	0.033*** (0.010)			0.058*** (0.016)		
RF (% Tax)		0.023** (0.010)			0.045*** (0.017)	
RF (% GDP)			0.033*** (0.010)			0.060*** (0.016)
GDP per capita	0.101 (0.100)	0.200* (0.109)	0.133 (0.100)	−0.035 (0.160)	0.053 (0.178)	0.021 (0.160)
Health expenditure	0.100*** (0.031)	0.090*** (0.031)	0.100*** (0.031)	0.028 (0.049)	0.014 (0.051)	0.028 (0.049)
FDI per capita	−0.006 (0.004)	−0.005 (0.004)	−0.006 (0.004)	−0.002 (0.006)	−0.003 (0.006)	−0.002 (0.006)
Household spending	−0.235*** (0.087)	−0.272*** (0.09)	−0.234*** (0.087)	0.014 (0.139)	0.070 (0.151)	0.016 (0.139)
OOP expenditure	0.056 (0.051)	0.053 (0.053)	0.056 (0.051)	−0.132 (0.082)	−0.159* (0.087)	−0.132 (0.082)
External expenditure	0.003 (0.007)	0.006 (0.007)	0.003 (0.007)	−0.001 (0.011)	0.001 (0.011)	−0.001 (0.011)
Adj *R*^2^	0.803	0.799	0.803	0.493	0.474	0.496
Observations	343	329	342	342	328	341
*N*	54	51	53	54	51	53

Columns [1]–[3] are for the under-five mortality rate (U5MR) and Columns [4]–[6] reported analogously for the maternal mortality ratio (MMR). RF is revenue foregone, health expenditure is government health expenditure per capita PPP (USD), household spending is in per capita terms, OOP expenditure is out-of-pocket payments as a share of current health expenditure, and external expenditure is development assistance for health per capita PPP (USD).

^*^ *P* < 0.1, **P < 0.05, ***P < 0.01.

The control variables generally match their *a priori* expectations, although mostly insignificant. Higher household spending is associated with improved child health outcomes, but not maternal health outcomes, through the mechanisms described above, whereby richer households can afford better health services, especially for their children, increasing their chances of survival. Government health expenditure per capita is, counterintuitively, associated with worse child health outcomes (there is no impact on maternal health). This shows that while the level of government health expenditure is a key determinant of health, the efficiency of such spending—and health system efficiency in general—is just as important.

### Instrumental variable results

Table 3 reports the IV results. Columns [1] and [2] are for the two-stage least squares (2SLS), while columns [3] and [4] are for the Lewbel (2012) method. The diagnostic tests reported in panel B of Table 2 confirm the validity of both IV techniques. The 2SLS results confirm the primary findings: revenue foregone is associated with worse child and maternal health outcomes. The coefficients on revenue foregone are also larger, suggesting that the baseline fixed effects may be downward-biased due to other unobserved time-varying factors. The heteroskedasticity-based Lewbel (2012) results also reaffirm the robustness of the primary findings: revenue foregone is associated with deteriorating health outcomes, also with larger coefficients.

**Table 3.** Tax expenditures and health outcomes, instrumental variables.

	2SLS		Lewbel (2012)	
	U5MR [1]	MMR [2]	U5MR [3]	MMR [4]
RF per capita	0.086*** (0.020)	0.087** (0.034)	0.046** (0.020)	0.097*** (0.037)
GDP per capita	0.046 (0.126)	−0.178 (0.179)	0.094 (0.107)	−0.057 (0.166)
Health expenditure	0.091** (0.044)	0.119* (0.066)	0.102*** (0.036)	0.034 (0.057)
FDI per capita	−0.005 (0.004)	0.001 (0.005)	−0.005 (0.003)	−0.000 (0.005)
Household spending	−0.280*** (0.094)	−0.059 (0.153)	−0.237*** (0.091)	0.010 (0.139)
OOP expenditure	−0.069 (0.070)	−0.093 (0.092)	0.056 (0.071)	−0.133 (0.083)
External expenditure	−0.021** (0.008)	−0.018 (0.015)	0.003 (0.010)	−0.002 (0.017)
Observations	252	250	343	342
*N*	36	35	54	54
K-*P* rk LM st. *P*-value	22.898 (0.000)	22.974 (0.000)		
K-P rk Wald *F*-statistic	10.786	10.872		
Hansen *J* statistic	1.519	0.696	24.697	28.236
Hansen *J P*-value	0.218	0.404	0.536	0.297

Columns [1] and [2] report the heteroskedasticity-robust results from the two-stage least squares (2SLS) estimation with two lags of the revenue foregone variable used as external instruments. The validity of the 2SLS technique is ascertained thus. First, an under-identification test, the Kleinbergen-Paap (K-P) rk LM statistic, to assess the relevance of the instruments, with a rejection of the null hypothesis indicating that the model is identified. Second, a weak-identification test to ascertain the relevance of the instruments. A rejection of the null of weak instruments occurs when the K-P rk F-statistic is greater than the Stock and Yogo (2002) critical values, or as a rule of thumb, when the K-P rk F-statistic is greater than 10. Third, the Hansen *J* test of over-identifying restrictions to ascertain the validity of the instrument set (i.e. that the instruments are orthogonal to the error term). The null hypothesis is that the instrument set is good and the model is correctly specified, a rejection of which casts doubt on the validity of the instruments. Columns [3] and [4] report the results from the Lewbel (2012) method using internally generated instruments, with the Hansen *J* test of over-identifying restrictions, again, confirming their validity.

^*^ *P* < 0.1, **P < 0.05, ***P < 0.01.

### Role of PFM quality

Table 4 shows that strong PFM curtails the adverse effects of tax expenditures on health outcomes. We analyse further by controlling for the level of PFM quality based on the average level of the CPIA variables over the sample period. We split countries between low and high PFM based on the third quartile of the variables (rather than the median) to: (i) focus on the countries with overwhelmingly stronger PFM and (ii) have a balanced number of countries in each sub-sample. Supplementary Table B2 shows that PFM quality, specifically the quality of public administration and efficiency of revenue mobilization, had a mediating impact on the tax expenditure variable only in the cluster of countries with the strongest PFM quality.

**Table 4.** Tax expenditures and health outcomes, PFM quality.

	U5MR			MMR		
	[1]	[2]	[3]	[4]	[5]	[6]
RF (PPP)	0.119** (0.048)	0.167*** (0.039)	0.267*** (0.065)	0.222*** (0.054)	0.151*** (0.048)	0.292*** (0.076)
RF (PPP)*CPIA1	−0.097** (0.043)			−0.203*** (0.049)		
RF (PPP)*CPIA2		−0.152*** (0.038)			−0.135*** (0.046)	
RF (PPP)*CPIA3			−0.210*** (0.054)			−0.242*** (0.063)
GDP per capita	−0.514*** (0.159)	−0.404** (0.158)	−0.527*** (0.170)	−0.870*** (0.179)	−0.862*** (0.196)	−0.814*** (0.201)
Health expenditure	0.005 (0.038)	−0.006 (0.037)	−0.003 (0.038)	−0.001 (0.042)	0.015 (0.046)	−0.017 (0.046)
FDI per capita	−0.008* (0.004)	−0.005 (0.005)	−0.006 (0.004)	−0.005 (0.005)	−0.004 (0.006)	−0.001 (0.005)
Household spending	−0.430*** (0.141)	−0.514*** (0.140)	−0.416*** (0.143)	0.006 (0.159)	0.001 (0.173)	−0.022 (0.170)
OOP expenditure	−0.143 (0.087)	−0.098 (0.084)	−0.073 (0.087)	0.003 (0.098)	0.040 (0.104)	−0.005 (0.102)
External expenditure	−0.075*** (0.016)	−0.069*** (0.015)	−0.073*** (0.015)	−0.061*** (0.018)	−0.059*** (0.019)	−0.062*** (0.019)
Adj *R*^2^	0.648	0.716	0.711	0.618	0.562	0.592
Observations	150	150	150	149	149	149
N	26	26	26	26	26	26

CPIA1, quality of public administration; CPIA2, transparency, accountability, and corruption in the public sector; CPIA3, efficiency of revenue mobilization.

^*^ *P* < 0.1, **P < 0.05, ***P < 0.01.

### Robustness analysis

First, alternative measures of revenue foregone. The findings are reported in Table 2: columns [2] and [3] for the under-five mortality rate and columns [5] and [6] for the maternal mortality ratio. The primary results remain robust to the alternative definitions of revenue foregone, with the effects still stronger on maternal mortality compared with under-five mortality. Second, splitting countries by level of development. Table 5 shows that tax expenditures are associated with worsening health outcomes only in LLMICs, with no significant effect for UMICs. Third, we include additional controls as described above. Supplementary Table B3 shows that the additional variables do not alter the primary findings, as increased tax expenditures are still associated with higher child and maternal mortality. Fourth, alternative methods (see the methods and justification above). The primary results remain robust to alternative estimators (see Supplementary Tables B4 and B5). Fifth, estimates show that there are no threshold or non-linear effects in the models. However, the effect of revenue foregone on maternal mortality is higher in countries with higher revenue foregone (see Supplementary Table B6).

**Table 5.** Tax expenditures and health outcomes, level of development.

	U5MR		MMR	
	(LLMICs)	(UMICs)	(LLMICs)	(UMICs)
RF per capita PPP	0.031*** (0.009)	0.024 (0.031)	0.056*** (0.012)	−0.002 (0.044)
GDP per capita	−0.038 (0.101)	−0.119 (0.197)	−0.072 (0.133)	−0.563** (0.284)
Health expenditure	0.039 (0.026)	0.359*** (0.084)	0.034 (0.034)	0.403*** (0.121)
FDI per capita	−0.004 (0.003)	−0.004 (0.008)	0.003 (0.004)	−0.009 (0.011)
Household spending	−0.245*** (0.093)	0.095 (0.194)	0.185 (0.122)	0.381 (0.279)
OOP expenditure	0.016 (0.058)	0.320*** (0.100)	0.247*** (0.076)	0.133 (0.144)
External expenditure	−0.011 (0.009)	0.021* (0.011)	0.015 (0.012)	0.045*** (0.015)
Adj *R*^2^	0.887	0.806	0.851	0.271
Observations	208	135	207	135
*N*	34	20	34	20

^*^ *P* < 0.1, **P < 0.05, ***P < 0.01.

## Discussion

We sought to ascertain the impact of revenue foregone (the direct cost of tax expenditures) on health outcomes, specifically the under-five mortality rate and maternal mortality ratio, in developing countries. The main analysis used revenue foregone per capita PPP as the primary independent variable, with revenue foregone % GDP and % tax revenue used for robustness. Irrespective of the measure of revenue foregone used, the results demonstrate a strong relationship between tax expenditures and health outcomes. Baseline analysis shows that an increase in revenue foregone per capita is associated with higher child and maternal mortality ratios, with the estimated coefficients larger for maternal mortality. The stronger effects on maternal mortality are potentially intuitive. The major causes of maternal mortality in developing countries are strongly linked to the availability of resources, which ultimately influences the availability and quality of care (most of which is preventive and linked to emergency obstetric care), hence more easily treatable or preventable. They, consequently, require relatively less greenfield health investments and interventions to handle. In resource-constrained settings, foregone revenue from tax expenditures can contribute more effectively to the pool of resources to combat those causes of maternal mortality linked to preventive care. The primary causes of child mortality, on the other hand, require considerable investments to handle. While some of the causes of child mortality also depend crucially on the availability and timeliness of preventive care, other more greenfield healthcare interventions (linked to curative care) are necessary to improve the survival of children and reverse any early damage. For example, investing in developing health infrastructure, reducing malnutrition, and its ancillary effects. All these interventions require significant resources, and foregone revenue may be just part of the total pool of resources needed for these interventions.

Country heterogeneity is also salient in the analysis. We found a significant relationship between tax expenditures and health outcomes only in LLMICs, with no effects in UMICs. This may not be surprising, given that UMICs have larger budget space compared with LLMICs. UMICs collect more taxes in absolute terms, hence have higher tax ratios and so can afford to lose more through tax expenditures: an assertion confirmed in Von Haldenwang et al. (2023). Since LLMICs collect less taxes on average, the revenue foregone represents a larger proportion of total tax collected, hence, a more ‘important’ financing option to those economies, hence the strong adverse effects on health outcomes in LLMICs. In addition, LLMICs typically have limited health insurance schemes (both compulsory and voluntary), much higher OOP, and are more susceptible to the corrosive effects of tax avoidance. All these contrive to make revenue foregone from tax expenditures a very important potential source of funding for health in LLMICs.

The level of PFM quality is included only as an intermediate factor in the analysis, and results show that increases in PFM quality mitigate the adverse impact of tax expenditures on health outcomes. We distinguish between high and low PFM quality using sub-sample analysis, splitting countries based on the third quartile. Those above are considered to have ‘high PFM’ and those below are considered low. Our findings on PFM and tax expenditures have two crucial implications. Firstly, they corroborate evidence reporting the importance of PFM through its direct impact on health outcomes (Tapsoba et al. 2024), improved PFM expanding budget space for health (Barroy and Gupta 2021), and strong PFM as a necessary prerequisite for tax expenditure evaluation and reporting (IMF 2022). Secondly, the findings add to the studies that show a relationship between factors eroding DRM and health outcomes, being conditional on institutional quality (Ortega et al. 2018, 2020).

Tax expenditures are popular since they do not require upfront budgetary disbursements and appropriations; they are not included in the budget process, and offer policymakers opportunities to neutralize legislative impediments to government spending (Dean 2012). Thus, they can occasionally be financed at lower political costs. The steep financial costs and risks to fiscal sustainability involved in granting them, however, cannot be ignored. The findings in this paper suggest that the adverse effects of tax expenditures on health outcomes may be quantitatively small, but that is unsurprising, given that the revenue foregone could have been invested in a variety of sectors, including education, social protection, and even infrastructure development. The findings in this paper can, thus, be seen as fundamentally contributory to the burgeoning literature on the social costs of tax expenditures (Aliu and Redonda 2024), analyses which will be useful in underpinning policy options for country-specific tax expenditure reform.

## Limitations and avenues for future research

The empirical analysis has some limitations. First, there is limited data on tax expenditures across countries. Many countries, especially developing ones like those in our sample, do not consistently provide tax expenditure reports, leaving analysis with a largely unbalanced panel of countries. Second, it would have been useful to control the models for key determinants that should inevitably contribute to the health service delivery and consequently to health improvement. These include primary healthcare financing (share in total health expenditures), health system inputs (e.g. the availability of drugs, health workforce, health infrastructure, and equipment) for which the available data were insufficient to perform econometric regressions based on the study sample.

The novelty of the research question introduces various avenues for further research. Generate a measure of theoretical fiscal capacity (revenue collected plus potential revenue from eliminating tax expenditures, i.e. revenue gain) and use it to capture the maximum possible revenue base. Including this as a primary explanatory variable allows comparison with findings that use ‘revenue foregone’ alone. If higher theoretical fiscal capacity is associated with improved health outcomes, but higher tax expenditures (revenue foregone) weaken health outcomes, this strengthens the interpretation that tax expenditures constrain budget space (for health). Likewise, analysing the impact of revenue foregone on health systems outputs (e.g. service delivery inputs such as immunization, service coverage index, and ANC coverage) is a fruitful avenue for further research.

## Conclusion

Over recent decades, tax expenditures have been at the centre of global policy discourse, with development partners and tax administrations taking various initiatives to inform the tax expenditure reform agenda. These efforts are warranted, given that tax expenditures constitute a steep fiscal cost for developing countries struggling to create fiscal space and still reeling from the effects of concurrent global crises. Within that context, reducing tax expenditures should free up useful government resources to invest in human development outcomes. Rigorous empirical analysis linking tax expenditures with development outcomes can complement the initiatives undertaken by development partners and tax administrations and contribute to the body of knowledge underpinning the fervour for tax expenditure reform. To that end, this paper analysed the impact of tax expenditures on child and maternal health outcomes in 55 developing countries covering the period from 2000 to 2022.

The findings show that increased tax expenditures are associated with worse child and maternal health outcomes, with much stronger effects (almost double) on maternal health outcomes. IV techniques confirm the primary results and permit a causal interpretation. The results also show that institutional quality, specifically PFM quality, mitigates the adverse effects of health outcomes (albeit in a reduced sample). In addition, we find the effects to be heterogeneous: tax expenditures are associated with worse health outcomes in low-income and lower middle-income countries.

## Supplementary Material

czaf079_Supplementary_Data

## Data Availability

The data used in this paper are available in the public domain and are easily accessible.

## Reflexivity statement

Our author group comprises one female and two male mid-career researchers of African descent, both of whom are based in Africa (Ouagadougou and Pretoria, respectively). The other author (HB) is based in Geneva, but constantly travels to developing countries for work. Two of the three authors have their PhDs in Health Economics (Y.T. and H.B.), while the other author has theirs in Development Economics (A.T.). The corresponding author (A.T.) is a Public Finance Specialist with extensive experience in tax policy analysis (especially quantitative analysis in cross-country contexts), focusing on tax expenditure analysis and evaluation in low- and middle-income countries, particularly in sub-Saharan Africa (SSA). The other authors (Y.T. and H.B.) have extensive experience in health financing options and challenges in low- and middle-income countries in Africa (including quantitative analysis), and H.B. also has huge experience in other developing regions. Thus, all the authors are economists with have strong experience with public finance and health systems within a developing country context, with these relatively diverse backgrounds crucial to the success of the collaboration. The female author (H.B.) is a senior researcher with extensive knowledge and expertise in the subject matter, and her experience was invaluable in guiding the two mid-career researchers (A.T. and Y.T.).
