# Supplementary material for: Expanding budget space to improve health outcomes in low- and middle-income countries: what role for tax expenditures?
Source: Health Policy Plan. 2025 Oct 22;41(2):127–38. doi: 10.1093/heapol/czaf079 (PMC12906764; doi:10.1093/heapol/czaf079)
Supplement: czaf079_Supplementary_Data [file czaf079_supplementary_data.docx]

**SUPPLEMENTARY MATERIALS**

**Appendix A – Data**

**A1 List of countries**

Albania, Argentina, Armenia, Benin, Bolivia, Brazil, Bulgaria, Burkina Faso, Burundi, Cabo Verde, Cameroon, Colombia, Congo, Dem. Rep., Costa Rica, Cote d’Ivoire, Dominican Republic, El Salvador, Eswatini, Gabon, Georgia, Ghana, Guatemala, Guinea, Honduras, India, Indonesia, Jamaica, Jordan, Kazakhstan, Kenya, Lesotho, Madagascar, Mali, Mauritania, Mauritius, Mexico, Mongolia, Morocco, Nicaragua, Niger, Pakistan, Papua New Guinea, Paraguay, Peru, Philippines, Rwanda, Senegal, South Africa, Sri Lanka, Tanzania, Togo, Tunisia, Turkey, Uganda.

LLMICs (34): Algeria, Benin, Bolivia, Burkina Faso, Burundi, Cabo Verde, Cameroon, Congo Dem. Rep, Cote d’Ivoire, Eswatini, Ghana, Guinea, Honduras, India, Jordan, Kenya, Lesotho, Madagascar, Mali, Mauritania, Mongolia, Morocco, Nicaragua, Niger, Pakistan, Papua New Guinea, Philippines, Rwanda, Senegal, Sri Lanka, Tanzania, Togo, Tunisia, Uganda.

UMICs (21): Albania, Argentina, Armenia, Brazil, Bulgaria, Colombia, Costa Rica, Dominican Republic, El Salvador, Gabon, Georgia, Guatemala, Indonesia, Jamaica, Kazakhstan, Mauritius, Mexico, Paraguay, Peru, South Africa, Turkey.

**Table A1.** Descriptive statistics of main variables, 2000-22

| **Variable** | **Obs.** | **Mean** | **Standard deviation** | **Max.** | **Min.** |
| --- | --- | --- | --- | --- | --- |
| Under-five mortality rate | 624 | 3.50 | 0.78 | 5.08 | 1.79 |
| Maternal mortality ratio | 594 | 4.85 | 1.61 | 7.02 | 1.61 |
| Revenue foregone per capita PPP | 624 | 5.04 | 1.52 | 9.59 | -0.64 |
| Revenue foregone (% GDP) | 620 | 0.85 | 0.96 | 2.58 | -3.52 |
| Revenue foregone (% tax revenue) | 584 | 2.80 | 1.00 | 4.85 | -2.13 |
| GDP per capita constant PPP | 626 | 8.74 | 0.88 | 10.25 | 6.42 |
| Domestic health expenditure per capita PPP | 595 | 4.87 | 1.36 | 7.35 | 0.83 |
| External health expenditure per capita | 545 | 2.24 | 1.38 | 5.43 | -4.08 |
| FDI per capita outflows, USD | 461 | 1.98 | 2.30 | 5.25 | -7.00 |
| Household consumption per capita, USD | 567 | 8.34 | 0.79 | 9.72 | 6.08 |
| Out-of-pocket expenditure (% current health expenditure) | 594 | 3.49 | 0.55 | 4.44 | 1.68 |
| CPIA1 | 286 | 3.14 | 0.65 | 4.50 | 1.50 |
| CPIA2 | 286 | 3.15 | 0.48 | 4.00 | 2.00 |
| CPIA3 | 286 | 3.59 | 0.39 | 4.50 | 2.05 |

CPIA1 = quality of public administration, CPIA2 = transparency, accountability and corruption in the public sector, CPIA3 = efficiency of revenue mobilisation. All variables, except the CPIA variables, are reported in natural logarithms.

**Table A2.** Examples of general tax expenditures

| **Tax type** | **Economic Function** | **Policy Objective** | **Beneficiaries** | **Examples** |
| --- | --- | --- | --- | --- |
| PIT | Exemptions^a^ | Incentivise employment in specific areas; promote savings; reduce burden on fringe benefits | Employees; retirees | Pension contributions; employer-provided health insurance |
|  | Deductions^b^ | Encourage socially desirable spending; reducing the tax burden | Mostly consumers (e.g., individuals with high education expenses, employees etc) | Medical expense deductions, mortgage interest deductions |
|  | Credits^c^ | Increase affordability of certain services; incentivise behaviour | Individuals or households | Child Tax Credit, Earned Income Tax Credit (EITC) |
|  | Preferential rates^d^ | Encourage investment in capital | Investors; high-income individuals | Lower rates for capital gains and qualified dividends |
| CIT | Exemptions | Attract investment in specific sectors and locations; support NGOs and other non-profits | Businesses, NGOs | Tax holidays for qualified companies in zones, income exemption for NGOs |
|  | Deductions | Promote business investment in underserved and/or rural areas; reduce effective tax rates | Businesses (especially capital-intensive ones) | Depreciation allowances, R&D expense deductions |
|  | Credits | Support targeted sectors or activities (e.g., research and development (R&D)) | Businesses in specific industries | R&D tax credit, green energy credit |
|  | Deferrals^e^ | Boost domestic production of key goods; incentivise reinvestment; improve cash flow | Businesses (typically large corporations) | Accelerated depreciation on capital investments; earnings abroad |
|  | Reduced rates | Promote industrialisation or regional development | Businesses (e.g., exporters, manufacturing firms) | Lower CIT rates in free trade zones |
| VAT | Exemptions | Reduce the costs of essential goods and services | Consumers (individuals and households); businesses | Exemptions of basic foodstuff, financial services etc |
|  | Reduced rates | Promote affordability of priority goods and services | Consumers (households, individuals, priority sectors) | Reduced rates on books, electricity, public transport |
| Excise Taxes | Exemptions | Support low prices for essential goods | Individuals and households | Fuel tax exemptions for farmers or public transport |
| Customs Duties | Exemptions | Support industrial policy or reduce import costs | Manufactures (importers); priority sectors | Duty-free imports of capital equipment |
|  | Reduced rates | Facilitate availability of critical imports | Businesses | Raw materials for domestic manufacturing |
| Property Tax | Exemptions | Encourage investment; encourage urban development | Businesses (i.e., developers); local businesses | Exemptions in enterprise zones; brownfield redevelopment exemptions |

The table provides examples of common general (or broadly defined) tax expenditures broken down by tax type, economic function, policy objective and beneficiaries. ^a^ Exclusions from the tax base, ^b^ amounts deducted from the tax base before applying the statutory tax rate, ^c^ amounts deducted from tax liability, ^d^ different (typically reduced) tax rates, ^e^ delays in paying the tax liability. PIT = personal income tax, CIT = corporate income tax, VAT = value added tax.

**Table A3.** Partial correlation matrix

|  | U5MR | MMR | RF PPP | GDPPC | GHEGGE | DGHEPC | FDIPC | HOUSEPC |
| --- | --- | --- | --- | --- | --- | --- | --- | --- |
| U5MR | 1 |  |  |  |  |  |  |  |
| MMR | 0.831^***^ | 1 |  |  |  |  |  |  |
| RF PPP | -0.5432^***^ | -0.5419^***^ | 1 |  |  |  |  |  |
| GDPPC | -0.6517^***^ | -0.6597^***^ | 0.7321^***^ | 1 |  |  |  |  |
| GHEGGE | -0.5707^***^ | -0.4834^***^ | 0.5935^***^ | 0.5534^***^ | 1 |  |  |  |
| DGHEPC | -0.5992^***^ | -0.5696^***^ | 0.6912^***^ | 0.8959^***^ | 0.6842^***^ | 1 |  |  |
| FDIPC | -0.4262^***^ | -0.3969^***^ | 0.395^***^ | 0.4979^***^ | 0.5169^***^ | 0.4984^***^ | 1 |  |
| HOUSEPC | -0.6316^***^ | -0.6742^***^ | 0.7807^***^ | 0.9711^***^ | 0.5962^***^ | 0.8645^***^ | 0.5176^***^ | 1 |
| OOPCHE | 0.5346^***^ | 0.3141^***^ | -0.5142^***^ | -0.5515^***^ | -0.5884^***^ | -0.6331^***^ | -0.4366^***^ | -0.5562^***^ |
| EXTPC | 0.1122 | 0.0695 | 0.1701 | -0.1069 | -0.0114 | -0.1332 | -0.2147^***^ | -0.0511 |
| GOV | 0.6597^***^ | 0.5031^***^ | -0.5528^***^ | -0.655^***^ | -0.5965^***^ | -0.5756^***^ | -0.5655^***^ | -0.6634^***^ |
| HOSPI | -0.4568^***^ | -0.4165^***^ | 0.3331 | 0.6719 | 0.0813 | 0.6149 | 0.1776^*^ | 0.6102^***^ |
| SANI | -0.8155^***^ | -0.8163^***^ | 0.707^***^ | 0.7717^***^ | 0.5186^***^ | 0.6942^***^ | 0.4834^***^ | 0.7841^***^ |
| ENROL | -0.8166^***^ | -0.7013^***^ | 0.5747^***^ | 0.8177^***^ | 0.5632^***^ | 0.76^***^ | 0.559^***^ | 0.7621^***^ |
| DENS | 0.282^***^ | 0.1685^*^ | -0.2454^**^ | -0.2559^***^ | -0.3742^***^ | -0.3833^***^ | -0.1445 | -0.1689^*^ |
| STUN | 0.7045^***^ | 0.5799^***^ | -0.5931^***^ | -0.6705^***^ | -0.4121^***^ | -0.6267^***^ | -0.4361^***^ | -0.6597^***^ |
| IMMU | -0.7048^***^ | -0.3797^***^ | 0.3437^***^ | 0.3516^***^ | 0.3527^***^ | 0.3284^***^ | 0.1865^*^ | 0.3036^***^ |
| CPIA 1 | 0.5387^**^ | -0.0177 | -0.3049 | 0.4117^*^ | -0.524^**^ | -0.4185^*^ | 0.0978 | 0.4819^**^ |
| CPIA 2 | -0.2515 | 0.0859 | 0.086 | 0.252 | 0.3679 | 0.4853^**^ | 0.0088 | -0.0361 |
| CPIA 3 | -0.683^***^ | -0.0824 | 0.5267^**^ | 0.0849 | 0.7231^***^ | 0.7819^***^ | 0.3405 | -0.2649 |
|  | OOPCHE | EXTPC | GOV | HOSPI | SANI | ENROL | DENS | STUN |
| OOPCHE | 1 |  |  |  |  |  |  |  |
| EXTPC | -0.0916 | 1 |  |  |  |  |  |  |
| GOV | 0.55^***^ | 0.0334 | 1 |  |  |  |  |  |
| HOSPI | -0.3167^***^ | 0.0705 | -0.3162^***^ | 1 |  |  |  |  |
| SANI | -0.4808^***^ | -0.0406 | -0.5937^***^ | 0.5242^***^ | 1 |  |  |  |
| ENROL | -0.699^***^ | -0.2023^**^ | -0.7533^***^ | 0.5183^***^ | 0.7108^***^ | 1 |  |  |
| DENS | 0.4271^***^ | 0.0228 | -0.0012 | -0.1693^*^ | -0.129 | -0.3305^***^ | 1 |  |
| STUN | 0.7201^***^ | -0.0475 | 0.6971^***^ | -0.5415^***^ | -0.732^***^ | -0.7605^***^ | 0.2807^***^ | 1 |
| IMMU | -0.4039^***^ | 0.0972 | -0.5244^***^ | 0.3222^***^ | 0.4785^***^ | 0.4819^***^ | -0.2784^***^ | -0.547^***^ |
| CPIA 1 | 0.5651^***^ | -0.369 | 0.0232 | -0.5407^**^ | -0.3176 | -0.476^**^ | 0.1828 | 0.1228 |
| CPIA 2 | -0.5035^**^ | 0.0987 | -0.8337^***^ | 0.4561^**^ | -0.6603^***^ | 0.5352^**^ | -0.3671 | -0.4982^**^ |
| CPIA 3 | -0.6909^***^ | 0.55^**^ | -0.9032^***^ | 0.503^**^ | -0.2992 | 0.7415^***^ | -0.6706^***^ | -0.8503^***^ |
|  | IMMU | | CPIA 1 | | CPIA 2 | | CPIA 3 | |
| IMMU | 1 | |  | |  | |  | |
| CPIA 1 | -0.5808^***^ | | 1 | |  | |  | |
| CPIA 2 | 0.4535^**^ | | 0.0434 | | 1 | |  | |
| CPIA 3 | 0.7362^***^ | | -0.2084 | | 0.7677^***^ | | 1 | |

*P*<0.1^*^, *P*<0.05^**^, *P*<0.01^***^. Note. GDPPC: GDP per capita, PPP USD. GHEGGE: Government health expenditure (% of domestic government expenditure). DGHEPC: Domestic government health expenditure per capita PPP (USD). FDIPC: FDI per capita outflows (USD). HOUSEPC: Household spending per capita (USD). OOPCHE: Out-of-pocket expenditure on health (% of current health expenditure). EXTPC: External health expenditures per capita PPP (USD). GOV: Governance index. HOSPI: Density of hospital beds. SANI: Share of people using at least basic sanitation services. ENROL: Secondary enrolment rate. DENS: Population density. STUN: prevalence of stunting in children under five. IMMU: Immunization coverage index. CPIA1: Quality of public administration, CPIA2: Transparency, accountability and corruption in the public sector, CPIA3: Efficiency of revenue mobilisation.

**Appendix B – Supplementary Analysis**

**Table B1.** First-stage regression for 2SLS

|  | U5MR  [1] | MMR  [2] |
| --- | --- | --- |
| Revenue foregone per capita PPP (1^st^ lag) | 0.452***  (0.119) | 0.452***  (0.119) |
| Revenue foregone per capita PPP (2^nd^ lag) | 0.178*  (0.090) | 0.178**  (0.090) |
| GDP per capita | 0.992  (0.867) | 0.992  (0.863) |
| Health expenditure | 0.198  (0.252) | 0.198  (0.251) |
| FDI per capita | -0.044  (0.030) | -0.044  (0.030) |
| Household spending | -1.027  (0.690) | -1.027  (0.687) |
| OOP expenditure | -0.138  (0.333) | -0.138  (0.331) |
| External expenditure | 0.075  (0.048) | 0.075  (0.048) |
| Observations | 252 | 250 |
| *N* | 36 | 35 |
| K-P rk LM st. (*p*-value) | 22.898 (0.000) | 22.974 (0.000) |
| K-P rk Wald *F*-statistic | 10.786 | 10.872 |
| Hansen *J* statistic | 1.519 | 0.696 |
| Hansen *J* *p*-value | 0.218 | 0.404 |

*P*<0.1^*^, *P*<0.05^**^, *P*<0.01^***^. The rest as in Table 2.

**Table B2.** Tax expenditures and health outcomes, strongest PFM quality

|  |  | U5MR |  |  | MMR |  |
| --- | --- | --- | --- | --- | --- | --- |
|  | [1] | [2] | [3] | [4] | [5] | [6] |
| RF (PPP) | 0.024  (0.015) | 0.019  (0.015) | 0.025  (0.016) | 0.001  (0.018) | 0.021  (0.018) | 0.018  (0.018) |
| RF (PPP)*CPIA1 | -0.217***  (0.074) |  |  | 0.024  (0.091) |  |  |
| RF (PPP)*CPIA2 |  | 0.010  (0.014) |  |  | -0.001  (0.016) |  |
| RF (PPP)*CPIA3 |  |  | -0.034*  (0.018) |  |  | -0.064***  (0.020) |
| GDP per capita | -0.566***  (0.158) | -0.458***  (0.173) | -0.487***  (0.177) | -0.796***  (0.195) | -0.933***  (0.208) | -0.792***  (0.204) |
| Health expenditure | 0.021  (0.037) | -0.011  (0.043) | 0.002  (0.041) | 0.002  (0.046) | 0.021  (0.052) | -0.007  (0.047) |
| FDI per capita | -0.008*  (0.004) | -0.004  (0.005) | -0.007  (0.005) | -0.004  (0.005) | -0.004  (0.006) | -0.004  (0.005) |
| Household spending | -0.364**  (0.142) | -0.446***  (0.148) | -0.478***  (0.150) | -0.049  (0.174) | 0.058  (0.178) | -0.110  (0.173) |
| OOP expenditure | -0.101  (0.088) | -0.147  (0.090) | -0.191**  (0.092) | -0.052  (0.108) | 0.003  (0.108) | -0.108  (0.105) |
| External expenditure | -0.076***  (0.016) | -0.074***  (0.016) | -0.079***  (0.016) | -0.069***  (0.019) | -0.064***  (0.020) | -0.071***  (0.019) |
| Adj R^2^ | 0.712 | 0.677 | 0.684 | 0.560 | 0.529 | 0.577 |
| Observations | 150 | 150 | 150 | 149 | 149 | 149 |
| N | 26 | 26 | 26 | 26 | 26 | 26 |

CPIA1 = quality of public administration, CPIA2 = transparency, accountability and corruption in the public sector, CPIA3 = efficiency of revenue mobilization. *P*<0.1^*^, *P*<0.05^**^, *P*<0.01^***^. The rest as in Table 2 (see explanation in the results section – “Role of PFM quality”).

**Table B3.** Tax expenditures and health outcomes, additional determinants of health

|  | U5MR | MMR |
| --- | --- | --- |
| RF per capita | 0.046***  (0.011) | 0.084*  (0.047) |
| GDP per capita | 0.224***  (0.096) | 0.653*  (0.360) |
| Health prioritization | 0.106**  (0.042) | 0.012  (0.102) |
| FDI per capita | -0.001  (0.004) | -0.012  (0.019) |
| Household spending | -0.346***  (0.114) | -1.592***  (0.372) |
| OOP expenditure | -0.183***  (0.049) | -0.069  (0.189) |
| Governance index | -0.103**  (0.061) | 0.244  (0.235) |
| Hospital beds | -0.005  (0.047) | 0.500***  (0.181) |
| Basic sanitation services | 0.475***  (0.179) | -0.908*  (0.483) |
| Secondary school enrolment | -0.225**  (0.086) | -.0773**  (0.308) |
| Population density | 0.508**  (0.211) | 1.045*  (0.612) |
| Child stunting | 0.329***  (0.042) |  |
| Immunization rate | -0.226***  (0.071) |  |
| Number of midwives |  | -0.116  (0.109) |
| Adj R^2^ | 0.948 | 0.606 |
| Observations | 138 | 93 |
| *N* | 27 | 24 |

*P*<0.1^*^, *P*<0.05^**^, *P*<0.01^***^. The rest as in Table 2.

**Table B4.** Tax expenditures and health outcomes, alternative estimators

|  | RE | | FGLS | |
| --- | --- | --- | --- | --- |
|  | U5MR  [1] | MMR  [2] | U5MR  [3] | MMR  [4] |
| RF per capita | 0.019*  (0.011) | 0.041**  (0.016) | 0.000  (0.008) | 0.025*  (0.014) |
| GDP per capita | -0.209**  (0.101) | -0.467***  (0.157) | -0.153***  (0.052) | -0.367***  (0.112) |
| Health expenditure | 0.055  (0.034) | -0.025  (0.053) | -0.195***  (0.017) | -0.252***  (0.040) |
| FDI per capita | -0.010**  (0.004) | -0.009  (0.006) | 0.002  (0.002) | 0.001  (0.004) |
| Household spending | -0.306***  (0.096) | -0.111  (0.148) | -0.316***  (0.057) | -0.257**  (0.102) |
| OOP expenditure | 0.037  (0.054) | -0.159*  (0.083) | -0.037  (0.037) | -0.245***  (0.058) |
| External expenditure | 0.005  (0.008) | 0.001  (0.021) | -0.000  (0.004) | 0.001  (0.009) |
| Observations | 343 | 382 | 336 | 335 |
| *N* | 54 | 54 | 47 | 47 |

*P*<0.1^*^, *P*<0.05^**^, *P*<0.01^***^. The rest as in Table 2.

**Table B5.** Tax expenditures and health outcomes, Driscoll and Kraay (1998)

|  | U5MR | | MMR | |
| --- | --- | --- | --- | --- |
|  | FE | RE | FE | RE |
| RF per capita | 0.033***  (0.010) | 0.019**  (0.009) | 0.058**  (0.021) | 0.041*  (0.020) |
| GDP per capita | 0.101  (0.090) | -0.209***  (0.060) | -0.035  (0.091) | -0.467***  (0.074) |
| Health expenditure | 0.100*  (0.038) | 0.055  (0.039) | 0.027  (0.076) | -0.025  (0.080) |
| FDI per capita | -0.005**  (0.002) | -0.010***  (0.005) | -0.002  (0.004) | -0.009  (0.006) |
| Household spending | -0.235***  (0.047) | -0.306***  (0.055) | 0.014  (0.141) | -0.111  (0.121) |
| OOP expenditure | 0.056  (0.073) | 0.037  (0.058) | -0.132  (0.079) | -0.159*  (0.078) |
| External expenditure | 0.003  (0.016) | 0.005  (0.019) | -0.001  (0.023) | 0.001  (0.025) |
| Observations | 343 | 343 | 342 | 342 |
| N | 54 | 54 | 54 | 54 |

*P*<0.1^*^, *P*<0.05^**^, *P*<0.01^***^. The rest as in Table 2.

**Table B6.** Tax expenditures and health outcomes, non-linear effects

|  | U5MR | | | MMR | | |
| --- | --- | --- | --- | --- | --- | --- |
|  | Quadratic form | Subsample regression based on RF per capita median value | | Quadratic form | Subsample regression based on RF per capita median value | |
|  |  | Countries with lower RF per capita | Countries with higher RF per capita |  | Countries with lower RF per capita | Countries with higher RF per capita |
| RF per capita | 0.018 | 0.042^***^ | 0.045^*^ | -0.037 | 0.043^***^ | 0.084^**^ |
|  | (0.67) | (4.13) | (1.72) | (-0.85) | (2.97) | (2.09) |
| RF per capita² | 0.002 |  |  | 0.011^**^ |  |  |
|  | (0.59) |  |  | (2.36) |  |  |
| GDP per capita | 0.155 | -0.575^***^ | 0.098 | 0.058 | -0.220 | 0.023 |
|  | (1.12) | (-4.07) | (0.67) | (0.35) | (-1.10) | (0.10) |
| Health expenditure | 0.010^***^ | 0.060^**^ | 0.429^***^ | 0.026 | 0.051 | 0.581^***^ |
|  | (3.23) | (2.39) | (5.85) | (0.54) | (1.43) | (5.18) |
| FDI per capita | -0.005 | -0.005 | -0.006 | -0.002 | 0.006 | -0.008 |
|  | (-1.49) | (-1.54) | (-0.80) | (-0.33) | (1.30) | (-0.72) |
| Household spending | -0.248^***^ | 0.112 | -0.237 | -0.064 | 0.158 | -0.530^**^ |
|  | (-2.77) | (0.93) | (-1.65) | (-0.45) | (0.92) | (-2.41) |
| OOP expenditure | 0.058 | 0.064 | 0.239^***^ | -0.121 | 0.151^*^ | 0.183 |
|  | (1.13) | (1.03) | (2.81) | (-1.48) | (1.71) | (1.41) |
| External expenditure | 0.004 | -0.004 | 0.017^*^ | 0.004 | -0.006 | 0.039^***^ |
|  | (1.54) | (-0.34) | (1.89) | (0.36) | (-0.36) | (2.82) |
| Adj R^2^ | 0.850 | 0.925 | 0.818 | 0.620 | 0.879 | 0.540 |
| Observations | 343 | 162 | 181 | 342 | 162 | 180 |
| *N* | 54 | 37 | 31 | 54 | 37 | 31 |

*P*<0.1^*^, *P*<0.05^**^, *P*<0.01^***^. The rest as in Table 2.
